# Genomic Prediction with Genotype by Environment Interaction Analysis for Kernel Zinc Concentration in Tropical Maize Germplasm

**DOI:** 10.1534/g3.120.401172

**Published:** 2020-06-01

**Authors:** Edna K. Mageto, Jose Crossa, Paulino Pérez-Rodríguez, Thanda Dhliwayo, Natalia Palacios-Rojas, Michael Lee, Rui Guo, Félix San Vicente, Xuecai Zhang, Vemuri Hindu

**Affiliations:** * Department of Agronomy, Iowa State University, Ames, IA 50011,; ^†^ International Maize and Wheat Improvement Center (CIMMYT), El Batan, Texcoco CP 56237, Mexico,; ^‡^ Colegio de Postgraduados, Department of Statistics and Computer Sciences, Montecillos, Edo. De México 56230, México,; ^§^ College of Agronomy, Shenyang Agricultural University, Shenyang, Liaoning 110866, China, and; ** Asia Regional Maize Program, International Maize and Wheat Improvement Center (CIMMYT), ICRISAT Campus, Patancheru, Hyderabad, Telangana 502324, India

**Keywords:** *Zea mays L*., genetics, breeding, zinc, prediction, GenPred, Shared data resources, Genomic Prediction

## Abstract

Zinc (Zn) deficiency is a major risk factor for human health, affecting about 30% of the world’s population. To study the potential of genomic selection (GS) for maize with increased Zn concentration, an association panel and two doubled haploid (DH) populations were evaluated in three environments. Three genomic prediction models, M (M1: Environment + Line, M2: Environment + Line + Genomic, and M3: Environment + Line + Genomic + Genomic x Environment) incorporating main effects (lines and genomic) and the interaction between genomic and environment (G x E) were assessed to estimate the prediction ability (*r_MP_*) for each model. Two distinct cross-validation (CV) schemes simulating two genomic prediction breeding scenarios were used. CV1 predicts the performance of newly developed lines, whereas CV2 predicts the performance of lines tested in sparse multi-location trials. Predictions for Zn in CV1 ranged from -0.01 to 0.56 for DH1, 0.04 to 0.50 for DH2 and -0.001 to 0.47 for the association panel. For CV2, *r_MP_* values ranged from 0.67 to 0.71 for DH1, 0.40 to 0.56 for DH2 and 0.64 to 0.72 for the association panel. The genomic prediction model which included G x E had the highest average *r_MP_* for both CV1 (0.39 and 0.44) and CV2 (0.71 and 0.51) for the association panel and DH2 population, respectively. These results suggest that GS has potential to accelerate breeding for enhanced kernel Zn concentration by facilitating selection of superior genotypes.

Malnutrition arising from zinc (Zn) deficiency is a major risk factor for human health affecting nearly 30% of the world’s population (Bouis and Saltzman 2017; Gannon *et al.* 2017). The problem is more prevalent in low-and middle income countries (LMICs), and is highly attributed to lack of access to a balanced diet, reliance on cereal-based diets and ignorance of good nutritional practices (Welch and Graham 2004). Several approaches, such as food fortification, diversification and supplementation have been tried to reduce Zn deficiency. However, in LMICs, these methods have not been entirely successful (Misra *et al.* 2004; Stein 2010).

Breeding maize for increased Zn concentration may offer some relief. The Zn-enriched varieties can be widely accessible, will not require continued investment once developed, and they remain after the initial successful investment and research (Govindan 2011). Recently, maize varieties with 15–36% more Zn were released in Guatemala and Colombia (Listman 2019). Nevertheless, increased breeding efforts are required to develop more Zn-enriched varieties for a diverse range of environments and management practices. Progress toward developing those varieties has mainly relied upon conventional plant breeding approach that is labor-intensive and time-consuming. However, with the recent advances in genomics, new methods for plant breeding such as genomic selection (GS) can be used to identify genotypes with enhanced Zn concentration more efficiently and rapidly.

Kernel Zn concentration is determined at the end of a plant’s life cycle, so GS can enable selection of promising genotypes earlier in the life cycle. This reduces the time and cost of phenotypic evaluation and may increase the genetic gain per unit time and cost (Heslot *et al.* 2015; Manickavelu *et al.* 2017; Arojju *et al.* 2019). The utility and effectiveness of GS has been examined for many different crop species, marker densities, traits and statistical models and varying levels of prediction accuracy have been achieved (de los Campos *et al.* 2009, 2013; Crossa *et al.* 2010, 2013, 2014; Jarquín *et al.* 2014; Pérez-Rodríguez *et al.* 2015; Zhang *et al.* 2015; Velu *et al.* 2016). Although the number of markers needed for accurate prediction of genotypic values depends on the extent of linkage disequilibrium between markers and QTL (Meuwissen *et al.* 2001), a higher marker density can improve the proportion of genetic variation explained by markers and thus result in higher prediction accuracy (Albrecht *et al.* 2011; Zhao *et al.* 2012; Combs and Bernardo 2013; Liu *et al.* 2018). Importantly, higher prediction accuracies have been obtained when genotypes of a population are closely related than when genetically unrelated (Pszczola *et al.* 2012; Combs and Bernardo 2013; Spindel and McCouch 2016).

Initially, GS models and methods were developed for single-environment analyses and they did not consider correlated environmental structures due to genotype by environment (G x E) interactions (Crossa *et al.* 2014). The differential response of genotypes in different environments is a major challenge for breeders and can affect heritability and genotype ranking over environments (Monteverde *et al.* 2018). Multi-environment analysis can model G x E using genetic and residual covariance functions (Burgueño *et al.* 2012), markers and environmental covariates (Jarquín *et al.* 2014), or marker by environment (M x E) interactions (Lopez-Cruz *et al.* 2015). This approach to GS can successfully be used for biofortification breeding of maize because multi-environment testing is routinely used in the development and release of varieties.

Modeling covariance matrices to account for G x E allows the use of information from correlated environments (Burgueño *et al.* 2012). Mixed models that allow the incorporation of a genetic covariance matrix calculated from marker data, rather than assuming independence among genotypes improves the estimation of genetic effects (VanRaden 2008). The benefit of using genetic covariance matrices in G x E mixed models is that the model relates genotypes across locations even when the lines are not present in all locations (Monteverde *et al.* 2018). GS models capable of accounting for multi-environment data have extensively been studied in different crops (Zhang *et al.* 2015; Cuevas *et al.* 2016, 2017; Velu *et al.* 2016; Jarquín *et al.* 2017; Sukumaran *et al.* 2017a; Monteverde *et al.* 2018; Roorkiwal *et al.* 2018). In those studies, incorporating G x E demonstrated a substantial increase in prediction accuracy relative to single-environment analyses.

Kernel Zn has been investigated in several quantitative trait loci (QTL) analyses in maize and each study has reported that Zn concentration is under the control of several loci. The phenotypic variation explained by those loci ranges from 5.9 to 48.8% (Zhou *et al.* 2010; Qin *et al.* 2012; Simić *et al.* 2012; Baxter *et al.* 2013; Jin *et al.* 2013; Zhang *et al.* 2017a; Hindu *et al.* 2018). A Meta-QTL analysis across several of those studies identified regions on chromosome 2 that might be important for kernel Zn concentration (Jin *et al.* 2013). Additionally, genomic regions associated with Zn concentration were recently reported in a genome-wide association study of maize inbreds adapted to the tropics (Hindu *et al.* 2018). Whereas some of the regions were novel, four of the twenty identified were located in previously reported QTL intervals.

A wide array of maize genetic studies has reported considerable effects of G x E interactions for kernel Zn concentration (Oikeh *et al.* 2003, 2004; Long *et al.* 2004; Chakraborti *et al.* 2009; Prasanna *et al.* 2011; Agrawal *et al.* 2012; Guleria *et al.* 2013). However, genotypes with high-Zn concentration have been identified in both tropical and temperate germplasm (Ahmadi *et al.* 1993; Bänziger and Long 2000; Brkic *et al.* 2004; Menkir 2008; Chakraborti *et al.* 2011; Prasanna *et al.* 2011; Hindu *et al.* 2018). Additionally, evaluation procedures for kernel Zn are labor-intensive, expensive and time-consuming (Palacios-Rojas 2018). To the best of our knowledge, no study has examined the predictive ability of GS methods that incorporate G x E for Zn concentration in maize. Within the framework of the reaction norm model (Jarquín *et al.* 2014), the potential of GS for Zn using maize inbreds adapted to tropical environments were assessed. The objectives of this study were; (i) to evaluate the prediction ability for Zn using an association mapping panel and two bi-parental populations evaluated in three tropical environments, (ii) to assess and compare the predictive ability of different GS models, and (iii) to examine the effects of incorporating G x E on prediction accuracy for Zn.

## Materials and Methods

### Zinc association mapping (ZAM) panel

The ZAM panel consists of 923 inbreds from maize breeding programs of the International Maize and Wheat Improvement Center (CIMMYT). The panel represents wide genetic diversity for kernel Zn concentration (Hindu *et al.* 2018).

### Bi-parental DH populations

From the ZAM panel, four inbreds with contrasting Zn concentration were selected and used to form two bi-parental (doubled haploid [DH]) populations. DH1 was derived from the F1 generation of a mating between CML503, a high-Zn inbred (31.21 μg/g) with CLWN201, a low-Zn inbred (22.62 μg/g). DH2 was derived from the F1 generation of a mating between CML465, another high-Zn inbred (31.55 μg/g) with CML451, a moderate-Zn inbred (27.88 μg/g). DH1 and DH2 were comprised of 112 and 143 inbreds, respectively.

### Experimental design and phenotypic evaluation

#### Zinc association mapping (ZAM) panel:

The ZAM panel was grown at CIMMYT research stations in Mexico, during the months of June through September and November through March at Agua Fria in 2012 and 2013, and Celaya in 2012. Plot sizes and the experimental designs (Hindu *et al.* 2018).

#### Bi-parental DH populations:

The DH populations were grown at CIMMYT research stations in Mexico; Celaya in 2014 and Tlaltizapan (18°41’N, 99° 07′ W; 962.5 m asl) in 2015 and 2017. In 2014 and 2015, both populations were evaluated in single-replication trials (Hindu *et al.* 2018). In 2017, a randomized complete block design (RCBD) with two replications was used. The rows were 2.5 m long and 75 cm apart and each genotype was grown in a single row plot. All plots were managed according to the recommended agronomic practices for each environment.

From the ZAM panel and each DH population, four to six plants in each plot were self-pollinated, hand-harvested at physiological maturity, hand-shelled and dried to a moisture content of 12.5%. The bulked kernels from each plot are considered a representative sample and were used in subsequent Zn analyses as described (Hindu *et al.* 2018).

### Genotypic data

Genomic DNA was extracted from leaf tissues of all inbred lines (ZAM panel and DH populations) using the standard CIMMYT laboratory protocol (CIMMYT, 2005). The samples were genotyped using the genotyping by sequencing (GBS) method at the Institute for Genomic Diversity, Cornell University, USA (Elshire *et al.* 2011; Crossa *et al.* 2013). The restriction enzyme ApeK1 was used to digest DNA, GBS libraries were constructed in 96-plex and sequenced on a single lane of Illumina HISeq2000 flow cell (Elshire *et al.* 2011). To increase the genome coverage and read depth for SNP discovery, raw read data from the sequencing samples were analyzed together with an additional ∼30, 000 global maize collections (Zhang *et al.* 2015).

SNP identification was performed using TASSEL 5.0 GBS Discovery Pipeline with B73 (RefGen_v2) as the reference genome (Elshire *et al.* 2011; Glaubitz *et al.* 2014). The source code and the TASSEL GBS discovery pipeline are available at https://www.maizegenetics.net and the SourceForge Tassel project https://sourceforge.net/projects/tassel. For each inbred, the pipeline yielded 955, 690 SNPs which were distributed on the 10 maize chromosomes. After filtering using a minor allele frequency of 0.05 and removing SNPs with more than 10% missing data, 181,889 (ZAM panel) and 170, 798 (bi-parental) SNPs were used for genomic prediction.

### Phenotypic data analysis

For the ZAM panel, broad-sense heritability *(H^2^)* across environments was estimated as:

H2=σG2σG2+σGE2l+σe2lr

where $\sigma_{G}^{2}$ is the variance due to genotype, $\sigma_{GE}^{2}$ is variance due to genotype x environment, $\sigma_{e}^{2}$ is the error variance, *l* is the number of environments and *r* is the number of replications using multi-environment trial analysis with R (META-R) (Alvarado *et al.* 2016). For the DH populations, variance components based on the genomic relationship matrix were computed using BGLR package as implemented in GBLUP (Pérez and de los Campos 2014). An estimate of narrow-sense heritability (${\hat{h}}^{2}$) for each DH population was calculated as:

$${\hat{h}}^{2}=\frac{{\hat{\sigma }}_{g}^{2}}{{\hat{\sigma }}_{g}^{2}+{\hat{\sigma }}_{g}^{2}}$$

where ${\hat{\sigma }}_{g}^{2}$ is an estimate of the additive genetic variance and ${\hat{\sigma }}_{e}^{2}$ is an estimate of the residual variance.

Correlation coefficients between Zn and environments, descriptive statistics and phenotypic data distribution using boxplots were generated in R (core Team 2018). Line means (genotypic values) for the ZAM panel were estimated as Best Linear Unbiased Estimators (BLUEs) with a random effect for replications nested within each environment. Raw data (values) were used for the DH populations.

### Statistical models

Genomic models used in this study were based on the reaction norm model which models the markers (genomic) by environment interaction (Jarquín *et al.* 2014). This model is an extension of the Genomic Best Linear Unbiased Predictor (GBLUP) random effect model, where the main effects of lines (genotypes), genomic, environments and their interactions are modeled using covariance structures that are functions of marker genotypes and environmental covariates.

In this study, environment is the combination of site and year (site-by-year) and the adjusted means (BLUES) to be used in the genomic prediction models are obtained by fitting the phenotypes $y_{ij}$ as:

$$y_{ij}=\mu +E_{i}+L_{j}+LE_{ji}+e_{ij},$$

this linear model represents the response of the *j*^th^ (*j* = 1,…,*J*) genotype/line tested in the *i*^th^ (*i* = 1,…,*I*) environment and $\left\{y_{ij}\right\}$ as the sum of an overall mean μ plus random environmental main effect $\left[E_{i}\overset{iid}{∼}N\left(0,\sigma_{E}^{2}\right)\right],$ the random genotype effect$\left[L_{j}\overset{iid}{∼}N\left(0,\sigma_{L}^{2}\right)\right]$, the random interaction between the *j^th^* genotype and the *i*^th^ environment $\left[LE_{ji}\overset{iid}{∼}N\left(0,\sigma_{LE}^{2}\right)\right]$ and a random error term $\left[e_{ij}\overset{iid}{∼}N\left(0,\sigma_{e}^{2}\right)\right]$. From this linear model, *N*(.,.) denotes a normal random variable, *iid* stands for independent and identically distributed responses and $\sigma_{E}^{2}$, $\sigma_{L}^{2}$, $\sigma_{LE}^{2}$, $\sigma_{e}^{2}$ are the variances for environment, genotype, genotype by environment and residual error, respectively. The model above does not allow borrowing of information among genotypes because the genotypes were treated as independent outcomes.

Thus, models used in this study were derived from the baseline model above by subtracting terms or modifying assumptions and/or incorporating genomics/marker information. A brief description of the genomic models used in this study are given below.

### M1. Environment + Line

This model is obtained by retaining the first three components from the baseline model (overall mean, random environment main effect and random line main effect) while their underlying assumptions remain unchanged.

$$y_{ij}=\mu +E_{i}+L_{j}+e_{ij}.$$ [1]

Here environments were considered as site-by-year combinations.

### M2. Environment + Line + Genomic

Another representation of the random main effect of line $L_{j}$ in the previous model is considering a linear combination between markers and their correspondent marker effects, $g_{j}=\overset{p}{\underset{m=1}{\sum }}x_{jm}b_{m}$, such that

$$y_{ij}=\mu +E_{i}+L_{j}+g_{j}+e_{ij}$$ [2]

where $b_{m}\overset{iid}{∼}N\left(0,\sigma_{b}^{2}\right)$ represents the random effect of the *m*^th^ (*m* = 1,…,*p*) marker, $x_{jm}$ is the genotype of the j^th^ line at the m^th^ marker and $\sigma_{b}^{2}$ its correspondent variance component.

Therefore, $g={\left({\text{g}}_{1},\ldots ,{\text{g}}_{J}\right)}^{\text{'}}$, is the vector of genetic effects, and follows a normal density with mean zero, and a co-variance matrix $Cov\left(g\right)=G\sigma_{g}^{2}$ with $G=\frac{XX^{’}}{p}$ being the genomic relationship matrix (Lopez-Cruz *et al.* 2015) that describes genetic similarities among pairs of individuals. In this model, the line effect $L_{j}$ is retained to account for imperfect information and model mis-specification because of potential imperfect linkage disequilibrium between markers and quantitative trait loci (QTL).

### M3. Environment + Line + Genomic + Genomic × Environment

This model accounts for the effects of lines $L_{j},\;\text{of}$ markers (genomic) $g_{j}$, of environments ($E_{i}$) and the interaction between markers (genomic) and the environment $\left(gE_{ji}\right)$. The model includes the interaction between markers (genomics) and the environment via co-variance structure (Jarquín *et al.* 2014). The model is as follows:

$$y_{ij}=\mu +E_{i}+L_{j}+g_{j}+gE_{ji}+e_{ij}$$ [3]

Where $gE_{ji}$ is the interaction between the genetic value of the j*^th^* genotype in the *i^th^* environment and $\mathrm{gE}=\left\{gE_{ji}\right\}∼N\left(0,\left(Z_{g}GZ_{g}^{'}\right)\#\left(Z_{E}Z_{E}^{'}\right)\sigma_{gE}^{2}\right)$, where $Z_{g}$ and $Z_{E}$ are the correspondent incidence matrices for the effects of genetic values of genotypes and environments, respectively,

$\sigma_{gE}^{2}$ is the variance component of gE and # denotes the Hadamard product (element-to-element product) between two matrices.

### Model assessment

Models were first fitted to the entire data set to estimate variance components using the R-package BGLR (de los Campos *et al.* 2010; de los Campos and Perez-Rodriguez 2016). The information generated from the full data analyses was not used as prior information for the cross-validation schemes (CV1 and CV2) used for assessing the prediction accuracy of the different models.

### Prediction accuracy assessment using cross-validation

Two distinct cross-validation schemes that mimic prediction problems that breeders may face when performing genomic prediction were used (Burgueño *et al.* 2012). One random cross-validation (CV1) evaluates the prediction ability of models when a set of lines have not been evaluated in any environment (prediction of newly developed lines). In CV1, predictions are entirely based on phenotypic records of genetically related lines. The second cross-validation (CV2) is related to incomplete field trials also known as sparse testing, in which some lines are observed in some environments but not in others. In CV2, the goal is to predict the performance of lines in environments where they have not yet been observed. In this study, CV2 mimics a situation where lines are evaluated in two environments but missing in the third environment. Thus, information from related lines and the correlated environments is used, and prediction assessment can benefit from borrowing information between lines within an environment, between lines across environments and among correlated environments.

In CV1 and CV2, a fivefold cross-validation scheme was used to generate the training and validation sets to assess the prediction ability for Zn within the ZAM panel and each DH population. The data were randomly divided into five subsets, with 80% of the lines assigned to the training set and 20% assigned to the validation set. Four subsets were combined to form the training set, and the remaining subset was used as the validation set. Permutation of five subsets taken one at a time led to five training and validation data sets. The procedure was repeated 20 times and a total of 100 runs were performed in each population. The average value of the correlations between the phenotype and the genomic estimated breeding values (GEBVs) from 100 runs was calculated for the ZAM panel, and each DH population for Zn in each environment and was defined as the prediction ability (*r_MP_*).

### Data availability

All models were fitted in R (core Team 2018) using the BGLR package (Pérez and de los Campos 2014). All phenotypic and genomic data can be downloaded from the link: http://hdl.handle.net/11529/10548331

## Results

### Descriptive statistics

**Figure 1:**
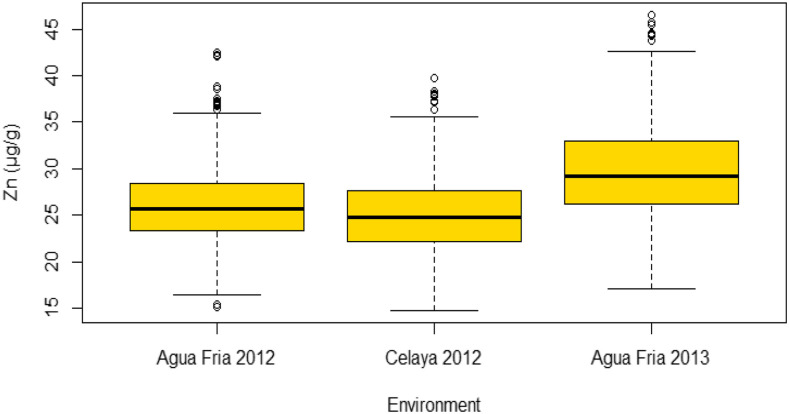
Box plot for kernel Zn (μg/g) in the ZAM panel in three environments (Agua Fria, 2012, Celaya, 2012 and Agua Fria 2013).

**Figure 2:**
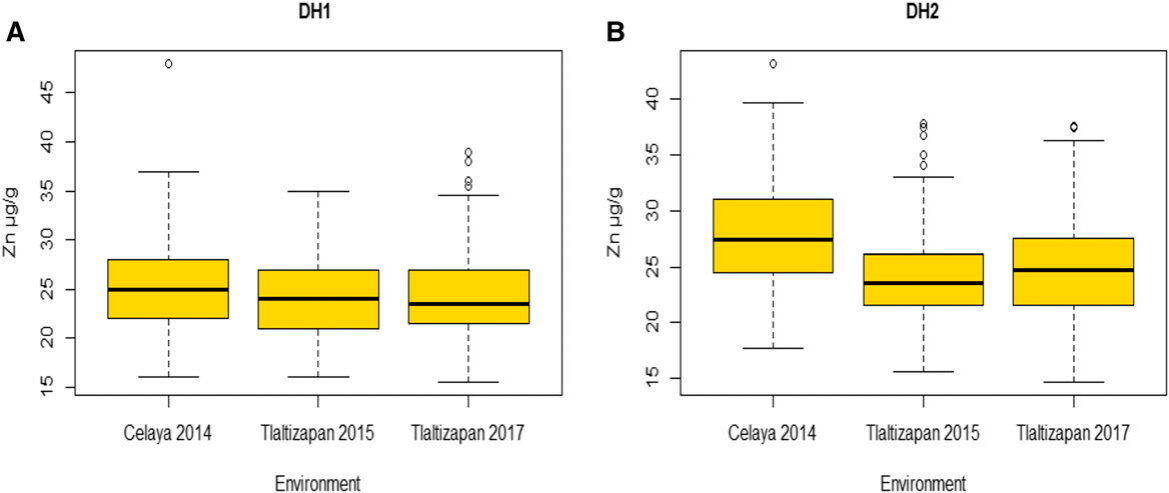
Box plot for kernel Zn (μg/g) for (A) DH1 and (B) DH2 in three environments (Celaya 2014, Tlaltizapan, 2015 and Tlaltizapan 2017).

Mean values of kernel Zn concentration were estimated for each environment and across environments (Tables 1 and 2). For the ZAM panel, kernel Zn ranged from 14.76 to 39.80 μg/g in Celaya 2012, 15.16 to 42.52 μg/g and 17.05 to 46.52 μg/g in Agua Fria 2012 and 2013, respectively (Figure 1). The highest mean (29.53 μg/g) for Zn was observed in Agua Fria 2013. DH1 had Zn values ranging from 16.00 to 48.00 μg/g in Celaya 2012, 16.00 to 35.00 μg/g in Tlaltizapan 2015 and 15.50 to 39.00 μg/g in Tlaltizapan 2017, while the respective values for DH 2 were 17.70 to 43.14 μg/g, 15.60 to 37.80 μg/g and 14.70 to 37.60 μg/g (Figures 2A and 2B). The highest means for Zn were observed in Celaya 2014 (25.38 μg/g) and 2017 (27.96 μg/g) for DH1 and DH2, respectively (Table 2). Across environments, *H^2^* for the ZAM panel was 0.85 (Table 1) and the $\hat{h^{2}}$for DH1 and DH2 were 0.83 and 0.76, respectively (Table 2). There were significant positive correlations between environments for Zn (Table 3), accounting for the moderate to high heritability estimates.

**Table 1:** Descriptive statistics for kernel Zn concentration for the ZAM panel grown in three environments

Population	Population size	Location	Mean ± SE (μg/g)	$\sigma_{G}^{2}$*^a^*	$\sigma_{GE}^{2}$*^a^*	*H^2^*
ZAM panel	923	Agua Fria 2012	26.15 ± 0.15	12.04	2.42	0.85
		Celaya 2012	25.06 ± 0.14			
		Agua Fria 2013	29.53 ± 0.16			
		Across	**26.94 ± 0.10**			

*H^2^=* Broad-sense heritability for Zn across environments.

a variance due to genotypes $\sigma_{G}^{2}$ and the interaction between genotypes and the environment $\sigma_{GE}^{2}$ significant at *P* < 0.001.

**Table 2:** Descriptive statistics for kernel Zn concentration for DH populations grown in three environments

Population	Population size	Location	Mean ± SE (μg/g)	${\hat{h}}^{2}$
DH1	112	Celaya 2014	25.38 ± 0.48	0.83
		Tlaltizapan 2015	24.01 ± 0.38	
		Tlaltizapan 2017	24.53 ± 0.37	
		Across	**24.65 ± 0.26**	
DH2	143	Celaya 2014	27.96 ± 0.39	0.76
		Tlaltizapan 2015	24.08 ± 0.33	
		Tlaltizapan 2017	24.64 ± 0.37	
		Across	**25.59 ± 0.22**	

$\hat{h^{2}}$ = Narrow-sense heritability for Zn across environments.

**Table 3:** Phenotypic correlation between environments for kernel Zn

	DH1	DH 2	ZAM Panel
*^a^*Env1 *vs.* Env2	0.62	0.46	0.63
*^a^*Env1 *vs.* Env3	0.58	0.29	0.66
*^a^*Env2 *vs.* Env3	0.62	0.45	0.61

Phenotypic correlation coefficients were significant at α = 0.001.

a DH populations; Env1, Env2 and Env3 = Celaya,2014, Tlaltizapan, 2017 and Tlaltizapan 2017, respectively.

a ZAM panel; Env1, Env2 and Env3= Agua Fria, 2012, Celaya, 2012 and Agua Fria 2013, respectively.

**Figure 3:**
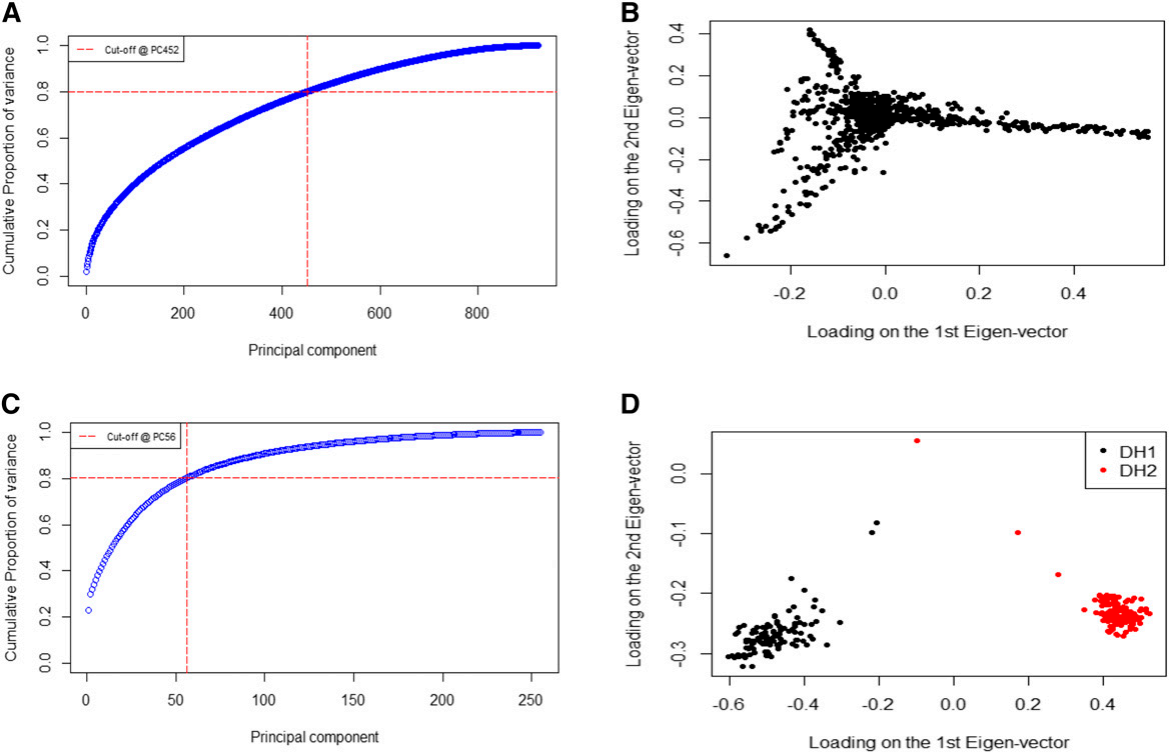
Scree plots (*A and C*) and loadings of the first two eigenvectors (*B and D*) of the covariance matrices derived from markers for the ZAM panel (A and B) and for the DH populations (*C and D*).

Principal component analysis for the ZAM panel suggested presence of a relatively diverse set of lines, and 452 principal components (PCs) were needed to explain 80% of the genotypes’ variance (Figures 3A and 3B). The first two principal components explained 3.85% of the total variance. For the DH populations first two eigenvectors separated the two groups (DH1 and DH2) and 56 principal components were needed to explain 80% of the genotypes’ variance (Figures 3C and 3D). The first two principal components explained 27.50% of the total variation for the DH populations.

### Estimates of variance components

Variance component estimates for all models were derived from the full-data analysis (Table 4). In the ZAM panel, variance components for M1 ranged from 16.18 to 7.01 with the main effect of environments explaining the largest proportion (46%). When marker information was incorporated (*i.e.*, M2 and M3), the estimated variance due to environments was reduced to 9.46 for M2 and 8.11 for M3. Inclusion of the interaction term (genomic x environment) reduced the residual variance component by ∼30%, from an estimated residual variance of 7.01 in M1 to 4.92 in M3 suggesting that some components of differences among genotypes cannot be fully captured by the main effects of markers and environments. Similar trends were observed in DH1 and DH2 except for M1 of DH1 where the main effect of lines accounted for a slightly higher proportion of the total variance (38%) than the main effects of environments (35%).

**Table 4:** Estimated variance components (estimate ± SD) and percentage of within-environment variance accounted for by each random effect

	Variance component estimate				Percentage of the within-environment variance*^a^*		
	Source	M1	M2	M3	M1	M2	M3
ZAM panel	*E*	16.18 ± 15.31	9.46 ± 6.43	8.11 ± 5.92			
	*L*	12.09 ± 0.71	2.44 ± 0.63	2.47 ± 0.64	63	13	13
	*G*	—	10.07 ± 1.11	10.03 ± 1.18	—	52	51
	*G x E*	—	—	2.13 ± 0.36	—	—	11
	*Residual*	7.01 ± 0.25	7.00 ± 0.25	4.92 ± 0.33	37	35	25
DH1	*E*	11.70 ± 9.67	7.72 ± 5.25	6.23 ± 4.58			
	*L*	12.77 ± 2.13	3.18 ± 1.09	2.96 ± 1.06	58	15	13
	*G*	—	9.67 ± 2.49	9.26 ± 2.54	—	44	42
	*G x E*	—	—	2.22 ± 0.76	—	—	10
	*Residual*	9.16 ± 0.88	8.95 ± 0.83	7.88 ± 0.89	42	41	35
DH2	*E*	12.37 ± 16.50	8.69 ± 6.13	7.26 ± 5.69			
	*L*	7.10 ± 1.30	2.36 ± 0.71	2.23 ± 0.71	39	10	9
	*G*	—	10.05 ± 2.94	9.41 ± 2.94	—	43	40
	*G x E*	—	—	2.90 ± 1.10	—	—	12
	*Residual*	11.07 ± 0.92	10.78 ± 0.86	9.31 ± 0.90	61	47	39

*E* =Environment, *L* = Line, *G* = genomic (marker information), *G x E* = genomic x environment.

a Relative to the total variance minus the variance due to main effect of the environment. The percentages of within-environment variance were computed without taking into account the variance of the environment.

The residual variance component values for all models of DH populations were slightly higher than those for the ZAM panel (Table 4), possibly because the populations were evaluated in single-replicated experiments at Celaya and Tlaltizapan (2014 and 2015, respectively). However, estimates from M3 of the DH populations suggest that ≥ 60 of the within-environment variability can be explained by main effects of lines, genomic (markers) and their interaction term. The proportions of within-environment variation explained by the interaction term were ≥ 10% for the ZAM panel and DH populations suggesting the importance of considering such interactions in genomic prediction models.

### Prediction ability in different populations

Cross-validated *r_MP_* values for kernel Zn were estimated for the ZAM panel and DH populations (Tables 5, 6 and 7). The average *r_MP_* values in CV1 were consistently lower than those in CV2, suggesting the importance of using information from correlated environments when predicting performance of inbred lines. The mean *r_MP_* values in CV1 and CV2 for the ZAM panel were 0.39 and 0.71, respectively (Table 5). For the DH populations, average *r_MP_* values were 0.53 for DH1-CV1, 0.44 for DH2-CV1 (Table 6), 0.70 for DH1-CV2 and 0.51 for DH2-CV2 (Table 7).

**Table 5:** Correlations (mean ± SD) between observed and genomic estimated breeding values for kernel Zn in the three environments for three GBLUP models for cross-validations CV1 and CV2 of the ZAM panel

		Prediction accuracy in CV1		
Population	Environment	M1*^a^*	M2	M3
	Agua Fria, 2012	−0.01 ± 0.04	0.33 ± 0.01	0.34 ± 0.02
ZAM panel (923)	Celaya, 2012	0.004 ± 0.04	0.43 ± 0.01	0.47 ± 0.01
	Agua Fria, 2013	−0.001 ± 0.03	0.34 ± 0.01	0.35 ± 0.01
	**Average**	**-0.001 ± 0.03**	**0.37 ± 0.01**	**0.39 ± 0.01**
		Prediction accuracy in CV2		
Population	Environment	M1*^a^*	M2	M3
	Agua Fria, 2012	0.71 ± 0.00	0.71 ± 0.00	0.72 ± 0.00
ZAM panel (923)	Celaya, 2012	0.64 ± 0.00	0.68 ± 0.00	0.72 ± 0.00
	Agua Fria, 2013	0.67 ± 0.00	0.67 ± 0.00	0.69 ± 0.01
	**Average**	**0.67 ± 0.00**	**0.69 ± 0.00**	**0.71 ± 0.00**

a Models: M1= Environment +Line; M2 = Environment + Line + Genomic; M3 = Environment + Line + Genomic + Genomic × Environment.

**Table 6:** Correlations (mean ± SD) between observed and genomic estimated breeding values for Zn in the three environments for three GBLUP models for cross-validation CV1 of DH populations

Population	Environment	Prediction accuracy in CV1		
		M1*^a^*	M2	M3
	Celaya, 2014	−0.05 ± 0.10	0.52 ± 0.04	0.51 ± 0.04
DH1	Tlaltizapan, 2015	−0.02 ± 0.12	0.52 ± 0.05	0.51 ± 0.05
	Tlaltizapan, 2017	−0.01 ± 0.10	0.56 ± 0.05	0.55 ± 0.05
	**Average**	**-0.03 ± 0.10**	**0.53 ± 0.04**	**0.52 ± 0.04**
	Celaya, 2014	0.05 ± 0.08	0.47 ± 0.03	0.50 ± 0.04
DH2	Tlaltizapan, 2015	0.03 ± 0.08	0.45 ± 0.03	0.45 ± 0.03
	Tlaltizapan,2017	0.04 ± 0.08	0.35 ± 0.03	0.35 ± 0.04
	**Average**	**0.04 ± 0.06**	**0.43 ± 0.03**	**0.44 ± 0.02**

a Models: M1= Environment +Line; M2 = Environment + Line + Genomic; M3 = Environment + Line + Genomic + Genomic × Environment.

**Table 7:** Correlations (mean ± SD) between observed and genomic estimated breeding values for Zn in the three environments for three GBLUP models for cross-validation CV2 of DH populations

Population	Environment	Prediction accuracy in CV2		
		M1*^a^*	M2	M3
	Celaya, 2014	0.67 ± 0.02	0.68 ± 0.02	0.68 ± 0.03
DH1	Tlaltizapan, 2015	0.70 ± 0.02	0.71 ± 0.02	0.70 ± 0.02
	Tlaltizapan, 2017	0.67 ± 0.02	0.70 ± 0.02	0.69 ± 0.02
	**Average**	**0.68 ± 0.01**	**0.70 ± 0.01**	**0.69 ± 0.01**
	Celaya, 2014	0.46 ± 0.016	0.53 ± 0.02	0.56 ± 0.02
DH2	Tlaltizapan, 2015	0.50 ± 0.020	0.55 ± 0.02	0.55 ± 0.02
	Tlaltizapan, 2017	0.40 ± 0.023	0.43 ± 0.02	0.43 ± 0.02
	**Average**	**0.45 ± 0.02**	**0.50 ± 0.01**	**0.51 ± 0.01**

a Models: M1= Environment +Line; M2 = Environment + Line + Genomic; M3 = Environment + Line + Genomic + Genomic × Environment.

In the ZAM panel, the highest values in CV1 (0.47) and CV2 (0.72) were obtained in Celaya and Agua Fria 2012 (Table 5). For the bi-parental populations, both under CV1 and CV2, higher *r_MP_* values were observed for DH1 compared to DH2. The highest values in CV1 (0.56) and CV2 (0.71) were observed in Tlaltizapan 2017 and 2015, all for DH1 (Tables 6 and 7). The consistently higher *r_MP_* values in CV1 and CV2 of DH1 could be attributed to the higher (0.58 to 0.62) correlation values between environments (Table 3).

### Prediction ability of different models

Comparing the *r_MP_* values obtained from each model, M1 had the lowest (-0.001, -0.03 and 0.04) accuracies in CV1 for the ZAM panel and DH populations (Tables 5 and 6). Those values were improved in CV2 because the predictions benefited from previous records (collected from other environments) of lines whose Zn values were being predicted. When M1 was expanded to M2 by adding the main effects of markers, the *r_M_*_P_ values at each environment and across environments were increased. For example, in CV1, M2, >100-fold increase in *r_MP_* values were observed for the ZAM panel and DH populations, and in CV2, M2, average *r_MP_* values increased by 2.98%, 2.94% and 11.11% for the ZAM panel, DH1 and DH2, respectively (Tables 5, 6 and 7).

The multi-environment model (M3), which includes the interaction between markers (genomic) and the environment $\left(gE_{ji}\right)$ gave higher prediction accuracy than single-environment models (M1 and M2). In CV1, mean *r_MP_* values increased from 0.37 (M2) to 0.39 (M3) for the ZAM panel and from 0.43 (M2) to 0.44 for DH2 (Tables 5 and 6). Similar trends were observed in CV2 for the ZAM panel and DH2 (Tables 5 and 7). However, in both CV1 and CV2 of DH1, incorporating $gE_{ji}$ did not improve *r_MP_* values for Zn (Tables 6 and 7). For CV1, M3, *r_MP_* values for Zn in individual environments ranged from 0.34 to 0.47 for the ZAM panel (Table 5), 0.51 to 0.55 for DH1 and 0.35 to 0.50 for DH2 (Table 6). For CV2, M3, those values ranged from 0.69 to 0.72 for the ZAM panel, 0.68 to 0.70 for DH1 and 0.43 to 0.56 for DH2 (Tables 5, 6 and 7).

## Discussion

Overall, moderate to high prediction ability values for kernel Zn were observed for the ZAM panel and DH populations. This could be attributed to the heritabilities observed for kernel Zn (Tables 1 and 2). Similar observations were reported for Zn concentration in wheat (Velu *et al.* 2016; Manickavelu *et al.* 2017). Higher predicted values with high accuracy for GS programs are expected for traits with moderate to higher heritability estimates (Combs and Bernardo 2013; Lian *et al.* 2014; Muranty *et al.* 2015; Saint Pierre *et al.* 2016; Manickavelu *et al.* 2017; Zhang *et al.* 2017b 2019; Arojju *et al.* 2019). Consistent with a study on Zn and iron (Fe) concentration in spring wheat, the prediction accuracies in this study are sufficient to discard at least 50% of the inbreds with low-Zn concentration (Velu *et al.* 2016).

Additionally, the moderate to high prediction accuracies reported in this study shows that GS can be used in maize breeding to improve kernel Zn concentration. Assuming two possible seasons of Zn evaluation per year, the predicted genetic gains can be estimated from prediction accuracies and genetic variances of the training populations. The genetic variances for the ZAM panel, DH1 and DH2 were 12.38, 12.20 and 14.88, and prediction accuracies were 0.71, 0.70 and 0.51, respectively. If the inbreds in each predicted population are ranked based on their predicted Zn values and the top 10% selected, then their expected average Zn values can be estimated from the proportion of inbreds selected, their respective training population genetic variances, prediction accuracies and the time interval for evaluating the lines. With reference to this, the expected average values of Zn are approximately 31 μg/g for the ZAM panel, 30 μg/g for DH1 and 27 μg/g for DH2. These averages are higher than the averages of the respective training populations (∼27 μg/g for the ZAM panel, ∼25 μg/g for DH1 and ∼26 μg/g for DH2) suggesting that the prediction accuracies achieved are sufficient to select at least 10% of the predicted inbreds with higher Zn concentration.

Data from both bi-parental populations and diverse collection of inbreds have been used for GS and based on cross-validation (CV), it has been established that prediction accuracies could also be affected by the relatedness between training and prediction sets (Habier *et al.* 2007; de Roos *et al.* 2009; Asoro *et al.* 2011; Daetwyler *et al.* 2013; Cericola *et al.* 2017; Crossa *et al.* 2017). In this study, average predicted accuracies were higher for CV1 of the bi-parental populations (0.53 for DH1 and 0.44 for DH2) compared to the ZAM panel (0.39). Higher predicted values in CV1 of the DH populations could be attributed to the closer relationship among DH lines in the training and prediction sets, maximum linkage disequilibrium (LD) between a marker and a QTL, and controlled population structure (Bernardo and Yu 2007; Albrecht *et al.* 2011; Zhang *et al.* 2015). In collections of diverse inbreds, prediction accuracy may depend on the ancestral relationships between the lines. So, in experiments using such collections of lines, prediction accuracies have been more variable than accuracies achieved using bi-parental populations (Spindel and McCouch 2016).

Cross-validation (CV) schemes are used in genomic prediction to estimate the accuracy with which predictions for different traits and environments can be made (Burgueño *et al.* 2012; Zhang *et al.* 2015; Saint Pierre *et al.* 2016; Velu *et al.* 2016; Sukumaran *et al.* 2017a, 2017b; Monteverde *et al.* 2018; Roorkiwal *et al.* 2018). In this study, two CV schemes (CV1- predicting the performance of newly developed lines, and CV2- predicting the performance of lines that have been evaluated in some environments, but not in others) were used. The utility of these schemes indicated that prediction values for newly developed lines (CV1) were generally lower (0.39 for the ZAM panel, 0.53 for DH1 and 0.44 for DH2) than the values for lines which have been evaluated in different but correlated environments (CV2; 0.71, 0.70 and 0.51 for the ZAM panel, DH1 and DH2, respectively). Such observations indicate the importance of using information from correlated environments when predicting the performance of inbred lines. However, selection of new lines without direct field testing, as simulated in CV1, may enhance the breeding process by replacing the time and labor-intensive field testing for Zn with genomic-estimated breeding values. But, the prediction accuracy values obtained may be lower such that the annual rate of genetic progress in a GS program is compromised (Burgueño *et al.* 2012). So, the ultimate decision of how a breeding scheme should be structured could depend on the compromise between the desired prediction accuracy and the generation interval (Burgueño *et al.* 2012).

Genotype by environment interaction is an important factor affecting kernel Zn concentration in maize and genomic prediction models that incorporate G x E may enhance the potential of GS for biofortification breeding. For different crop species and traits, genomic prediction models which incorporated G x E achieved higher prediction accuracies in both CV1 and CV2 schemes relative to models which did not include G x E (Burgueño *et al.* 2012; Guo *et al.* 2013; Jarquín *et al.* 2014; Lopez-Cruz *et al.* 2015; Zhang *et al.* 2015; Monteverde *et al.* 2018). In this study, the impact of modeling G x E variance structures for multi-environment trials was investigated and results indicated that the average predicted values from M3 (G x E model) were higher (0.39 and 0.44 for CV1 and 0.71 and 0.51 for CV2) than the values from M2 (non-G x E; 0.37 and 0.43 for CV1-M2, 0.69 and 0.50 for CV2-M2) for the ZAM panel and DH2. These findings agree with those reported on Zn concentration in wheat (Velu *et al.* 2016), providing evidence that incorporating G x E in GS models can enhance their power and suitability for improving maize for kernel Zn concentration. Conversely, the average predicted values for CV1 and CV2 of DH1 were higher in M2 (0.53 and 0.70) than in M3 (0.53 and 0.69). Except for differences in population size (112 lines *vs.* 143 lines), this was unexpected since DH1 and DH2 were grown in the same environments.

The gains in prediction accuracies for the GS model that accounted for G x E were dependent on the correlation between environments and CV method used. In this study, the phenotypic correlations between environments were all positive (ranging from 0.58 to 0.62 for DH1, 0.29 to 0.46 for DH2 and 0.61 to 0.66 for the ZAM panel). Such correlations can be exploited using multi-environment models to derive predictions that use information from across both the lines and environments (Burgueño *et al.* 2012). For instance, although the phenotypic correlations between environments for DH2 were positive (0.29 to 0.46), the lowest average prediction value (0.51) for CV2 was observed for this population. This was expected because CV2 uses phenotypic information from genotypes which have already been tested; hence, effectively exploiting the correlations between environments (Burgueño *et al.* 2012; Jarquín *et al.* 2014; Crossa *et al.* 2015; Pérez-Rodríguez *et al.* 2015; Saint Pierre *et al.* 2016; Monteverde *et al.* 2018). However, for CV1, the information between environments could only be accounted for through the genomic relationship matrix (Monteverde *et al.* 2018). Hence, the gains in CV1 may likely attribute to more accurate estimate of environment-specific marker effects (Guo *et al.* 2013). In contrast, when multiple environments are weakly correlated, prediction accuracies from across environment analyses can be negatively affected relative to prediction accuracies within environments (Bentley *et al.* 2014; Wang *et al.* 2014; Spindel and McCouch 2016). Thus, before designing a GS experiment, identifying correlated environments where environments can differ in terms of site, year or season in which data were collected is of great interest (Spindel and McCouch 2016).

However, the prediction accuracy values were of lower quality when genomic predictions were conducted across populations. For instance, when the ZAM panel was used as the training population, prediction accuracies for DH1, DH2 and DH1+DH2 were 0.15, -0.10 and 0.09, respectively. When DH1 and DH2 were used as a training and prediction set for each other, prediction accuracies were 0.08 and 0.16 (Unpublished data). These prediction accuracies are considerably lower than those reported in this study and the differences may be attributed to: (i) weak genetic relationships between the training and prediction population sets and (ii) different methods of analysis because the prediction accuracies reported in this study were partly achieved by modeling the random-effects environment structure to account for G x E while for the unpublished data, the random-effects environment structure of G x E was not included.

The ability to predict kernel Zn concentration using high-throughput SNP markers including G x E interactions creates an opportunity for efficiently enhancing Zn concentration in maize breeding programs. For instance, during early generations of a breeding program, GS can be utilized to identify genotypes with favorable alleles when numbers of progenies and families are large. This could potentially reduce the resource-intensive evaluation process and advancement of false-positive progenies (Velu *et al.* 2016). Coupled with advances in technologies for assessing Zn, plant scientists can more rapidly measure Zn concentration in maize kernels using the energy dispersive x-ray fluorescence (XRF) assays (Guild *et al.* 2017). Thus, with more validations and model refinements, GS can potentially accelerate the breeding process to enhance Zn concentration in maize for a wider range of environments.
